# The Prevalence and Distribution of Neurodegenerative Compound-Producing Soil *Streptomyces* spp.

**DOI:** 10.1038/srep22566

**Published:** 2016-03-03

**Authors:** Anna L. Watkins, Arpita Ray, Lindsay R. Roberts, Kim A. Caldwell, Julie B. Olson

**Affiliations:** 1 Department of Biological Sciences, University of Alabama, Tuscaloosa, Alabama

## Abstract

Recent work from our labs demonstrated that a metabolite(s) from the soil bacterium *Streptomyces venezuelae* caused dopaminergic neurodegeneration in *Caenorhabditis elegans* and human neuroblastoma cells. To evaluate the capacity for metabolite production by naturally occurring streptomycetes in Alabama soils, *Streptomyces* were isolated from soils under different land uses (agriculture, undeveloped, and urban). More isolates were obtained from agricultural than undeveloped soils; there was no significant difference in the number of isolates from urban soils. The genomic diversity of the isolates was extremely high, with only 112 of the 1509 isolates considered clones. A subset was examined for dopaminergic neurodegeneration in the previously established *C. elegans* model; 28.3% of the tested *Streptomyces* spp. caused dopaminergic neurons to degenerate. Notably, the *Streptomyces* spp. isolates from agricultural soils showed more individual neuron damage than isolates from undeveloped or urban soils. These results suggest a common environmental toxicant(s) within the *Streptomyces* genus that causes dopaminergic neurodegeneration. It could also provide a possible explanation for diseases such as Parkinson’s disease (PD), which is widely accepted to have both genetic and environmental factors.

Members of the filamentous bacterial genus *Streptomyces* are ubiquitous in soils and are well known producers of medically and agriculturally useful secondary metabolites. These nonessential metabolites are thought to promote the survival of the producing organism but are not directly involved in its growth, development, or reproduction, and include compounds such as antibiotics and toxins. Several classes of toxins produced by *Streptomyces* spp., including proteasome inhibitors (reviewed by)^1^ and mitochondrial complex I inhibitors^2^,^3^, have been recently receiving additional attention due to their potential role as environmental toxicants in human diseases^4^,^5^,^6^,^7^.

A metabolite(s) produced by *Streptomyces venezuelae* caused age- and dose-dependent degeneration of all neurons in the nematode model organism, *Caenorhabditis elegans*, with dopaminergic neurons selectively degenerating faster. Human SH-SY5Y neuroblastoma cells also degenerated in culture upon dose-dependent exposures^5^. The *S. venezuelae* metabolite caused cell death, in part, through decreased ATP production, modulation of mitochondrial complex I, and increased ROS^7^,^8^. We further determined that the metabolite induces disruptions in protein homeostasis, glutathione-tractable α-synuclein toxicity, and ubiquitin proteasome system activity. These activities are epistatically regulated by loss-of-function of the PARK6 homologue, *pink-1*^8^. These phenotypes were not exclusive to neurons, but occurred in all cells of the worm. However, because of the high energy requirements of neurons and muscle cells, obvious phenotypes were observed in these cell types^5^,^7^,^8^.

These data suggest that mitochondrial dysfunction from environmental exposure to *Streptomyces* metabolites may be a factor in cellular toxicity. The previous studies focused on a single species of *Streptomyces* (*S. venezuelae*). Here, an evaluation of naturally occurring strains was performed to assess whether soils supported robust streptomycete communities with the capability to cause dopaminergic neurodegeneration.

The degeneration of dopaminergic neurons is a hallmark of Parkinson Disease (PD), the second most prevalent neurodegenerative disorder. PD affects more than 1% of the population over age 65, increasing to 4-5% in people over age 85. Familial PD represents only a small percentage (~10%) of known cases, with the remaining disease thought to be due to a combination of interactions among environmental and intrinsic genetic factors. Twin studies indicated that environmental influences are critical to disease onset and appear pivotal to sporadic causality^9^. A greater incidence of PD has been associated with a rural lifestyle and farming as a profession^10^,^11^. Exposure to insecticides (e.g., rotenone)^12^,^13^,^14^,^15^,^16^,^17^, fungicides (e.g., maneb)^15^,^16^,^18^ and herbicides (e.g., paraquat)^15^,^16^,^17^,^18^,^19^ have been linked to PD; however, these exposures do not account for all associations between environmental risk and disease^10^,^20^. We hypothesize that exposure to bacterial metabolites produced by soil streptomycetes could be an environmental factor associated with neurodegeneration.

Soils harbor large and diverse microbial communities that can be affected by a variety of environmental conditions. Numerous studies have shown that substantial changes to the chemical composition of soil, often due to human use of the land, impact the resident microbial communities (e.g.,)^21^,^22^,^23^,^24^. Because PD is more prevalent in individuals with rural lifestyles, we wanted to evaluate the impact of land use on the diversity of resident soil streptomycetes and assess their ability to produce neurodegenerative metabolite(s). Soil properties, including pH, texture, and organic content, were measured for each sample to determine their impact on the diversity of *Streptomyces* present. A subset of the 1509 *Streptomyces* isolates recovered from soils under three land uses (agricultural, undeveloped, urban) were evaluated for their ability to cause dopaminergic neurodegeneration in *C. elegans*. We have previously established the utility of *C. elegans* for evaluation of genetic and environmental factors that impact dopaminergic neurodegeneration^25^,^26^,^27^,^28^; these worm models have been correlative to results obtained in mammalian systems^29^. Additionally, BOX-PCR, a fingerprinting method targeting repetitive, intergenic bacterial sequences, was used to assess the genomic diversity of all isolates. Our data showed that in natural populations, 28.3% (51/180) of genomically unique soil *Streptomyces* spp. isolated from diverse land uses in Alabama caused *C. elegans* dopaminergic neurons to degenerate. These results suggest that there could be a common environmental toxicant(s) within the *Streptomyces* genus that causes dopaminergic neurodegeneration.

## Results

### Differences in isolation of *Streptomyces* by land use and physiography

**Figure 1:**
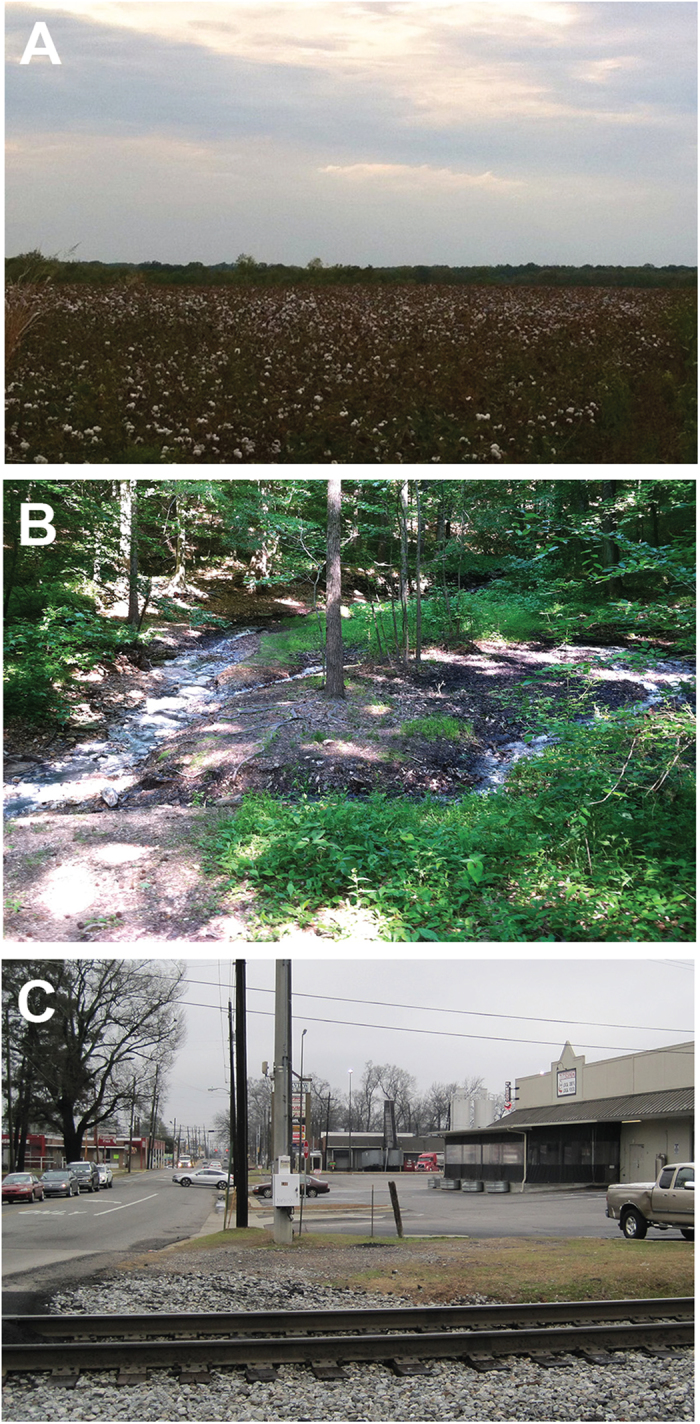
Representative images of land use soil sample collection sites. (**A**). Agricultural soils were collected from between rows of crops, such as from this cotton field. (**B**). Soils from undeveloped lands were also collected; samples taken from these locations usually required the removal of leaf litter prior to soil sampling. (**C**). Urban soils originated from small areas of land likely to be impacted by human activities and pollution.

**Figure 2:**
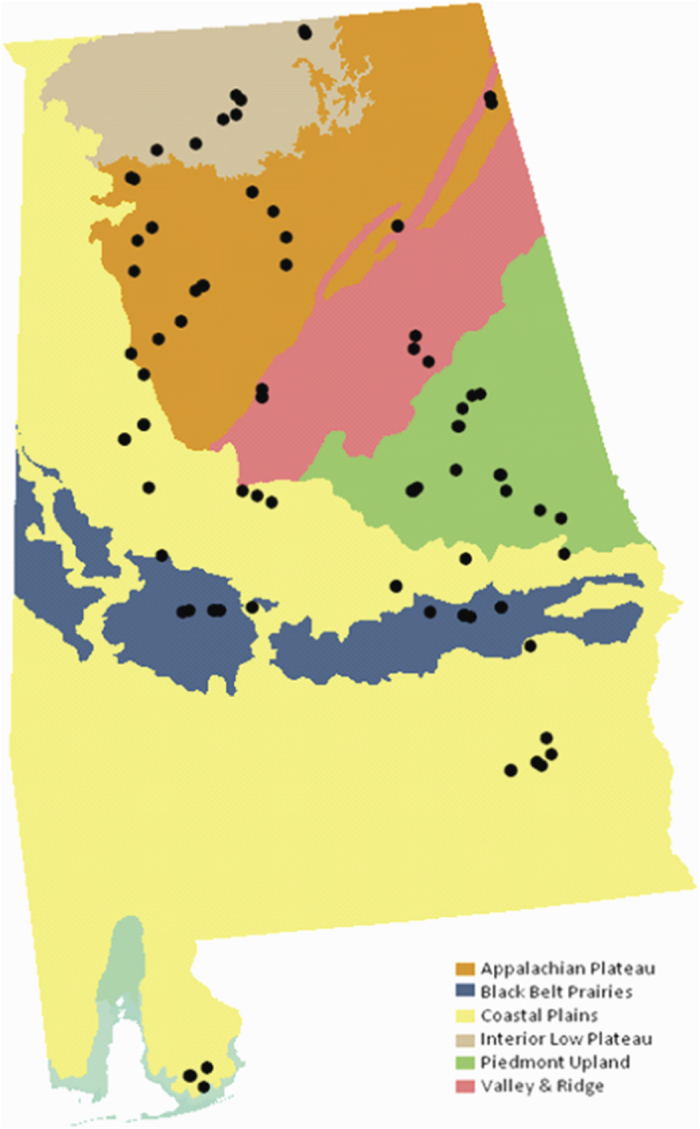
Soil sampling locations within the state of Alabama. Colors represent the physiographic provinces and data points (•) indicate sampling locations. Map courtesy of the University of Alabama Cartography Lab.

Samples were collected from soils under different land uses (e.g., agriculture, undeveloped, urban) across the state of Alabama (Fig. 1). For study purposes, agricultural soils were collected from lands used for cultivation of a plant species for consumption by humans or livestock, or retail. Land that supported substantial plant growth and appeared unused for any purpose was considered undeveloped. The presence of multiple tree species and significant undergrowth indicated that the land was not currently being utilized for agricultural purposes, nor had it been in recent years. Developed, or urban, lands included property near human populations, exclusive of lawns, which were likely to be impacted by pollution through exhaust and/or industrial applications. To represent the topographical and geologic diversity of the state, which affects the overlying soils, agricultural, undeveloped, and urban soil samples were collected and examined from each of the major physiographic provinces within Alabama (Fig. 2). Physiographic provinces are areas characterized by terrain texture, rock type and geologic structure with specific geomorphology or landforms different from adjacent provinces; Alabama lies at the confluence of five physiographic provinces (Coastal Plains, Piedmont Upland, Valley and Ridge, Cumberland Plateau, Interior Low Plateau). The Black Belt Prairies are a section of the Coastal Plains province. In total, 85 soil samples were obtained, with 27 from agricultural uses, 33 from undeveloped areas, and 25 from urban environments (Table 1).

**Figure 3:**
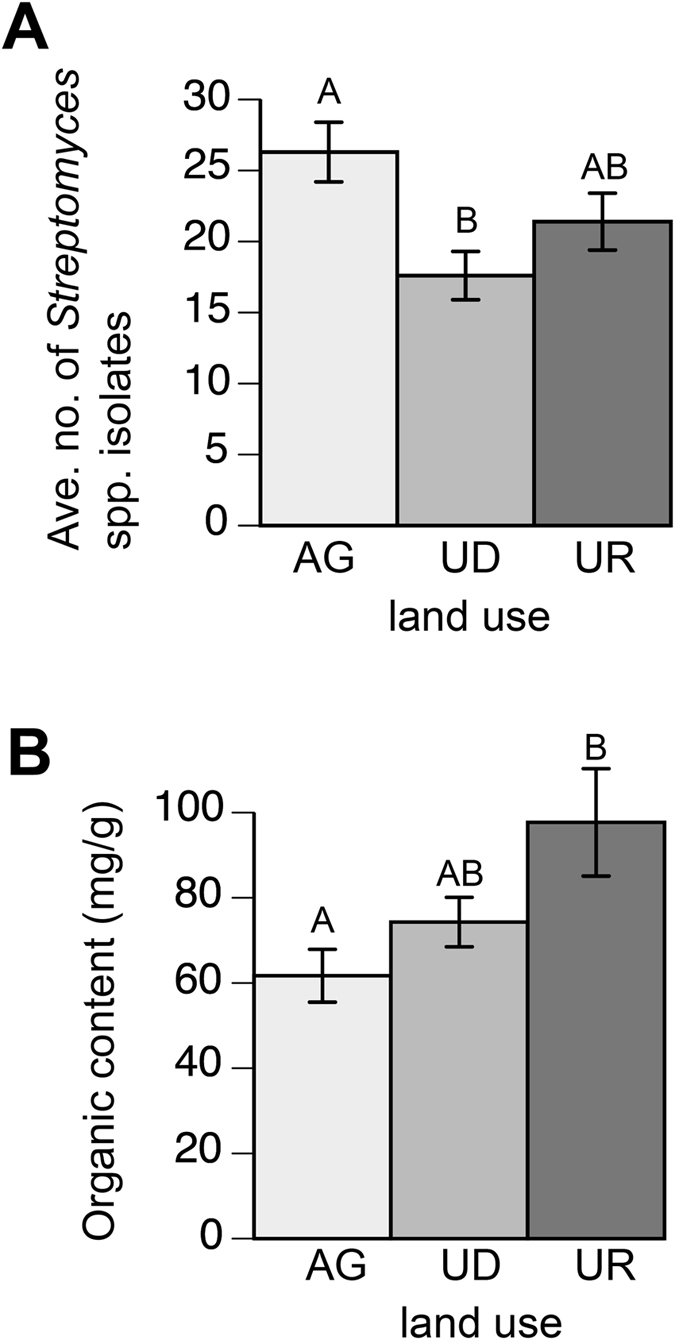
Characterization of *Streptomyces* spp. isolates. (**A**). A comparison of the average number of isolates obtained from dilutions of 0.25 g of agricultural (AG), undeveloped (UD) and urban (UR) soils. Statistical significance (p < 0.01) is shown between letters (ANOVA with Tukey’s HSD). Columns with the same letter are not significantly different from one another. Bars indicate the standard error of the mean (**B**). Organic matter content (mg/g) comparison among soils from AG, UD, and UR land uses. Statistical significance (p < 0.01) is shown between letters (ANOVA following a log conversion with Tukey’s HSD).

Cultivation of *Streptomyces* spp. from soils under different land uses varied, with a significant difference found in the number of isolates obtained between agricultural and undeveloped soils (one-way analysis of variance [ANOVA] with Tukey’s Honestly Significant Difference [HSD], p < 0.01; Fig. 3A). No significant differences in the isolation of *Streptomyces* spp. were found across physiographic provinces (p=0.905; Table 1). A total of 1509 isolates from 85 unique soil samples were characterized in this study, with an estimated range of 1.8 × 10^6^ to 4.2 × 10^8^ streptomycetes per gram of soil (Supplemental Table 1). On average, 24.1 ± 10 isolates were recovered from 0.25 g of soil used for agriculture compared to 15.1 ± 9.2 and 20.0 ± 9.1 isolates from 0.25 g of undeveloped and urban soils, respectively. There were no significant differences in isolation of streptomycetes among the 3 cultivation media employed (mannitol-soy, starch casein, and an environment specific agar; Chi-square, p = 0.43). The amount of organic matter (one-way ANOVA following a log conversion with Tukey’s HSD, p < 0.01; Fig. 3B) in the soils and soil pH (Kruskal-Wallis, p < 0.01) were significantly different across land use. A significant but weak positive correlation also existed between soil pH and isolation of *Streptomyces* (p < 0.05; r = 0.259). Soil pH differed significantly between agricultural and urban soils (Mann-Whitney, p < 0.01) and between undeveloped and urban soils (Mann-Whitney, p < 0.01). Unsurprisingly, across all land use patterns, soil pH significantly correlated with the amount of soil organic matter (SOM; Spearman’s correlation, p < 0.05). The greatest difference in SOM was found between agricultural and urban soils (Fig. 3B). No significant differences were found between land use and soil texture based on sand, silt or clay content (one-way ANOVA following a log conversion).

### Genomic diversity of *Streptomyces* isolates

**Figure 4:**
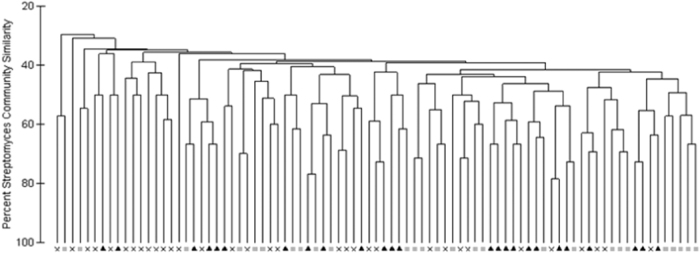
Cluster analysis of the *Streptomyces* composite fingerprints from each soil sample. Analyses of similarity found no statistical differences between land uses. As indicated on the X-axis, X = undeveloped soil samples; black triangles = urban soil samples; grey squares = agricultural soil samples.

Within dendrograms based on the similarity of genomic banding patterns generated during BOX PCR, isolates from the various land uses did not cluster (data not shown). Analysis of composite fingerprint patterns for each soil also showed no significant clustering by land use (Analysis of Similarity [ANOSIM], p > 0.05; Fig. 4). Based on banding patterns, of the 1509 isolates, only 112 (7.4%) were considered clones of other isolates. Thus, *Streptomyces* spp. possessing diverse genomes were recovered from soils exhibiting a broad range of pH values, organic matter content, and textures.

### *C. elegans* exposure to *Streptomyces* spp. supernatants

**Figure 5:**
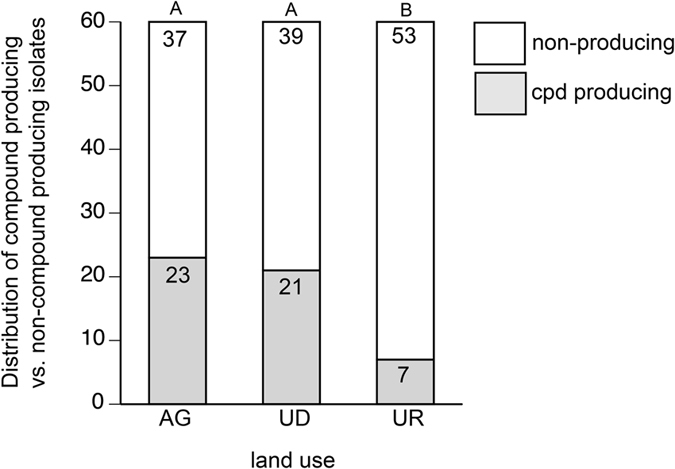
Significantly more *Streptomyces* spp. capable of producing a neurodegenerative compound were isolated from agricultural and undeveloped soils than from urban soils. n = 60 for each land use. Columns with the same letters are not significantly different from one another (Fisher’s exact test, p < 0.01).

*Streptomyces* isolates cultured from soils under each land use (agricultural, undeveloped, urban) were selected for analysis in the *C. elegans* dopaminergic model, with the locations representing all of the physiographic provinces within the state. The isolates from these locations were inoculated into SYZ broth for growth, which is conducive for the production of secondary metabolites. However, approximately half of the *Streptomyces* spp. inoculated did not grow in this medium, possibly due to the salt concentration. This limitation impacted both the number of isolates that we were able to test in the neurodegeneration assay and the physiographic distribution of the tested isolates. However, sixty isolates per land use were successfully grown and the filtered supernatants used for assessing neurodegeneration in a *C. elegans* model system. Of the isolates tested, 28.3% (51 of the 180 strains) were capable of producing neurodegeneration in the dopaminergic neurons of *C. elegans* (one-way ANOVA followed by a Bonferroni correction, p < 0.05). There was variability in the distribution of compound-producing *Streptomyces* across land uses (Fig. 5). Specifically, there were more isolates with neurodegenerative activity from agricultural and undeveloped soils compared to urban soils (Fisher’s exact test, p < 0.01). Similar percentages of compound producing strains were observed in agricultural (38.3%; 23 of 60 isolates) and undeveloped (35.0%; 21 of 60) soils while urban soils yielded a lower percentage of compound-producing strains (11.6%; 7 of 60). While it would be intriguing to further differentiate the neurodegenerative potential of *Streptomyces* spp. isolated from different physiographic provinces, too few isolates per physiographic province were tested for reliable statistical analyses. However, questions regarding compound-producing isolates and soil texture, pH, and organic content were addressed. No significant correlations between the prevalence of compound production and these characteristics were identified.

**Figure 6:**
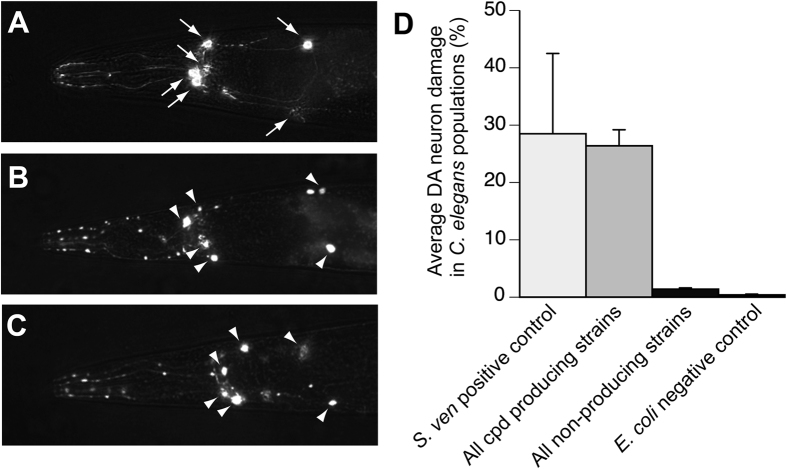
Exposure to environmental isolates of *Streptomyces* spp. resulted in dopaminergic neurodegeneration in *C. elegans*. (**A–C**). Representative images of *C. elegans* (strain BY200) expressing GFP specifically in the six anterior dopaminergic (DA) neurons. In all images, arrows show intact dopaminergic neuron cell bodies. Arrowheads indicate areas where dopaminergic neurons have degenerated. (**A**) An example of normal, intact, DA neurons that have not degenerated following 12 days exposure to negative control, *E. coli.* (**B**,**C**). *C. elegans* where all six DA neurons have degenerated following 12 days exposure to the *S. venezuelae* positive control (**B**) or a compound-producing *Streptomyces* sp. isolate (**C**). (**D**) Exposure to environmental isolates of *Streptomyces* spp. can cause significant neurodegeneration in populations of *C. elegans*.

An unequivocal advantage of *C. elegans* is that detailed analyses of neurons are achievable. There are precisely six dopaminergic neurons within the anterior region of the worm that consistently display degenerative characteristics (Fig. 6A). We examined these six neurons in *C. elegans* for changes in the cell bodies as well as the neuronal processes for normal appearance vs. degenerative changes (Fig. 6B,C). If a dopaminergic neuron displayed an abnormality (cell body loss, blebbing or missing neuronal processes), it was scored as degenerating and the total number of damaged neurons was recorded and compared to controls.

**Figure 7:**
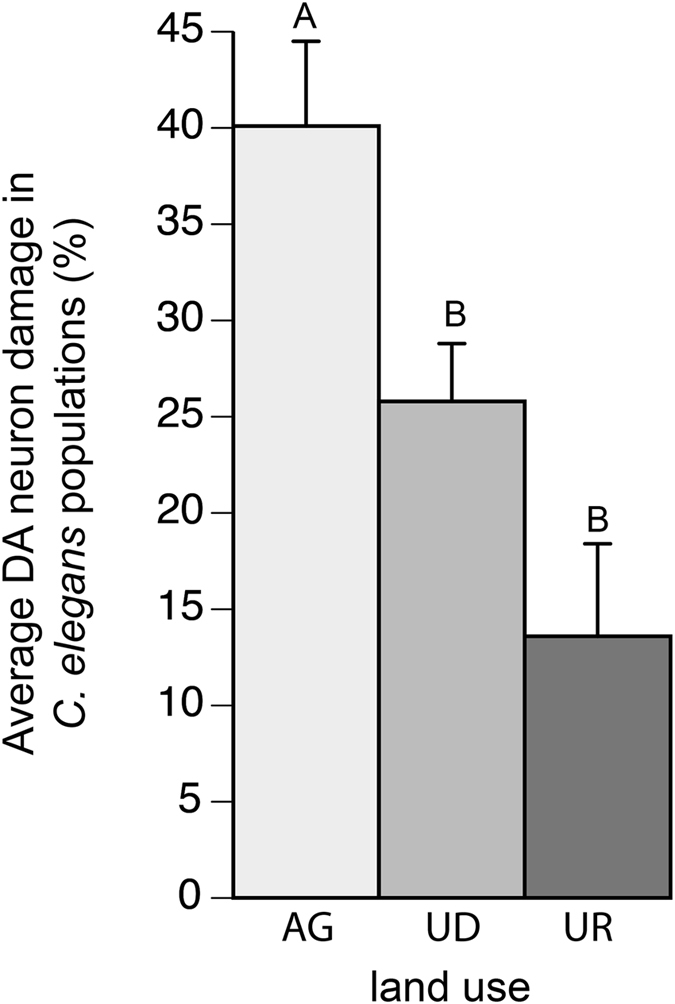
*Streptomyces* spp. isolated from agricultural soils caused significantly more dopaminergic neurodegeneration of individual neurons in *C. elegans* than isolates from undeveloped and urban soils. Columns with the same letters are not significantly different from one another (ANOVA, p < 0.05).

Using the degeneration of *C. elegans* dopaminergic neurons from exposure to *S. venezuelae* supernatant as a positive control^5^, 28.5% of individual dopaminergic neurons were found to be degenerated (Fig. 6D). Similarly, exposure to the supernatants of compound-producing soil *Streptomyces* spp. resulted in the degeneration of 26.4% of dopaminergic neurons. In contrast, exposure to the supernatants of non-compound producing isolates did not cause significant neurodegeneration, as only 1.4% of neurons, on average, degenerated in these nematodes. These data were comparable to the 0.4% neuron degeneration observed when animals were exposed to *E. coli* supernatants, the negative control. Interestingly, the amount of individual dopaminergic neuron damage in *C. elegans* exposed to compound-producing strains varied between land uses. *C. elegans* exposed to supernatants of isolates from agricultural soils caused more individual neurons to degenerate compared to strains isolated from undeveloped (one-way ANOVA, p < 0.05; Fig. 7) and urban (p < 0.05; Fig. 7) soils. All dopaminergic neuron counts from compound-producing soil streptomycetes were non-significantly different from the compound-producing *S. venezuelae* positive control and significantly different from the *E. coli* negative control (p < 0.01).

To determine if strains capable of producing the neurodegenerative compound(s) were similar, the genomic banding patterns obtained from BOX PCR of the tested *Streptomyces* isolates (n=180) were evaluated using Gel Compar II. No clustering of species capable of producing the neurodegenerative compound(s) was apparent within the dendrograms (data not shown). Instead, compound-producing species were distributed throughout the dendrograms, suggesting that strains capable of producing the neurodegenerative compound(s) exhibit substantial genomic diversity.

## Discussion

Exposure to the metabolites produced by *Streptomyces venezuelae* resulted in degeneration of all neuron classes in a *C. elegans* model and human neuroblastoma cells^5^. This led us to question the prevalence of neurodegenerative compound-producing *Streptomyces* spp. in natural environments. Based on the elevated incidence of PD, a neurodegenerative disease associated with rural living, drinking well water, and farming as a profession (e.g.,)^10^,^11^, we hypothesized that more neurodegenerative metabolite-producing organisms would be isolated from rural soils (e.g., agricultural, undeveloped) than urban soils.

*C. elegans* was ideal for these studies because it is a rapidly cultured transparent organism with an experimentally accommodating lifespan. Despite its evolutionary distance from humans, the 302 neurons of this genetically invariant hermaphroditic nematode retain many hallmarks of mammalian neuronal function including ion channels, neurotransmitters, vesicular transporters, receptors, and synaptic components^30^. The six anterior dopamine-producing neurons in this animal were readily evaluated for neurodegenerative changes using GFP as a fluorescent indicator. The use of *C. elegans* to rapidly evaluate the impact of environmental exposures on the survival of dopaminergic neurons is one of the strengths of this model system.

Notably, 28.3% of the *Streptomyces* isolates produced a compound(s) that caused dopaminergic neurodegeneration in *C. elegans*. The similarity in the percentages of neurodegenerative metabolite producing strains from agricultural (38.3%) and undeveloped (35.0%) soils compared to urban (11.6%) soils supported our hypothesis. Interestingly, significantly fewer *Streptomyces* spp. were isolated from undeveloped soils compared to agricultural soils, suggesting that land use alters the soil environment for *Streptomyces*. Additionally, strains isolated from agricultural soils resulted in more individual dopaminergic neuron damage than strains recovered from undeveloped or urban soils, indicating that the production of the neurodegenerative compound(s) is variable and may change with environment (Fig. 7). Although Basilio *et al*.^31^ reported differences in metabolite production from isolates obtained from environments with differing pH and salinity, the edaphic properties examined in this study (pH, organic matter content, and texture) did not appear to influence the distribution of producing strains. Alternately, the media used for isolation of the *Streptomyces* may select for strains with this capacity, as media composition affects bacterial recovery^32^ and metabolite production^33^,^34^,^35^. Obviously, much work remains to be done to address the potential impact of this neurodegenerative metabolite and its possible role as a bacterial toxicant in the etiology of PD.

This study utilized culture-dependent methods, which are generally thought to recover only a small portion (<1%) of the resident microorganisms, indicating that greater diversity of streptomycetes is likely in the soils examined. However, this approach was selected as it allowed us to specifically examine isolates for the production of the neurodegenerative compound, which, at present, cannot be assessed using culture-independent molecular techniques. It is possible that certain genera, including the aerobic, spore-forming *Streptomyces* with the ability to utilize a wide variety of carbon sources, are more amenable to cultivation than other genera. For example, a culture-dependent investigation of Antarctic soils indicated that the majority of actinobacteria isolated were *Streptomyces* (>80%), although they also found that the isolates were most closely related to culturable species while the phylotypes identified using cultivation-independent methods were related to uncultured species^36^. More work is needed to evaluate the full complement of resident *Streptomyces* spp. for the capacity to produce compounds that cause dopaminergic neurodegeneration, especially as this genus has been shown to account for ~6% of soil microbial libraries^37^.

Although BOX PCR was found to be effective in discriminating between closely related *Streptomyces* species in another study^38^, evaluation of our BOX banding patterns indicated that there was no uniformity within producing and non-producing strains (as determined by our *C. elegans* bioassay). However, Davelos *et al*.^39^ reported poor correspondence between 16 S rRNA gene grouping and BOX PCR fingerprints, suggesting that the fingerprinting method may reflect the instability of the *Streptomyces* genome, especially in the divergent ‘arms’ (reviewed by)^40^. In our samples, isolates producing a compound that caused dopaminergic neurodegeneration were widespread, implying that the genes for compound production may have been moved between hosts via lateral gene transfer (LGT). Much debate exists regarding the occurrence and ecological significance of LGT in streptomycetes, with some biosynthetic gene clusters suggested to be transferred between species laterally^41^,^42^,^43^,^44^ while others are more likely vertically transmitted^45^.

Of the 1509 *Streptomyces* isolates recovered in this study, 92.5% (1397 isolates) exhibited unique genomic banding patterns by BOX-PCR methods. As many important biosynthetic and metabolic genes have been found to be plasmid-encoded within this genus, the entire genetic information for an isolate was examined, including any plasmids. All colonies demonstrating the distinctive wrinkled, powdery morphology of *Streptomyces* were designated for isolation, eliminating potential selection bias by the investigators. Additionally, the predicted densities of streptomycetes per gram of soil (roughly 10^6^–10^8^ cells) are equivalent to, or in excess of, previous estimates derived from cultivation based analyses^39^,^46^,^47^,^48^,^49^, suggesting that the genus was adequately sampled and highlighting the extensive genomic diversity. As many natural products research programs were discontinued due to the high rediscovery rate of known compounds, these data support the prediction that less than 10% of bioactive metabolites have been discovered from this genus^50^,^51^ and indicate that members of *Streptomyces* possess extremely diverse genomes that likely include many novel biosynthetic gene clusters overlooked in previous phenotypic studies.

In this study, genomically diverse *Streptomyces* isolates were obtained from all samples but similar communities were not detected within soils under the same land use (Fig. 4) or from the same physiographic province (data not shown). We isolated more *Streptomyces* strains from agricultural soils compared to undeveloped soils but found no significant difference in streptomycete isolation from urban and undeveloped soils. However, as discussed by Lauber *et al*.^52^ soil microbial communities often reflect the characteristics of the soil in which they reside, indicating that land use alone cannot be used to predict community composition, as edaphic properties are not necessarily consistent within soils from a specific land use. Due to the exceedingly high level of genomic diversity discovered within the cultivated *Streptomyces* strains, it was not possible to evaluate what environmental factors affected their distribution. However, similar to results published by Lauber *et al*.^53^ more *Streptomyces* strains were isolated from soils with neutral to slightly alkaline pH. This result was somewhat unexpected, as a number of previous studies had portrayed *Streptomyces* as an acidophilic genus commonly recovered from soils exhibiting lower pH (e.g.,)^54^,^55^,^56^. Regardless, it is interesting that none of the strains appeared to have substantial localized populations, minimizing the possibility for cell-to-cell communication via quorum sensing approaches. However, the small amount of soil used for cultivation and the known spatial heterogeneity of soil populations may have influenced the recovery of similar strains. Even with these limitations, it appears that substantial genetic variation exists within members of the *Streptomyces*, reinforcing the utility of the genus for continued discovery of novel natural products.

## Conclusions

The role of bacterial metabolites, particularly toxins, in human health has been well established (reviewed by)^57^,^58^. However, considerably less is known about the role of environmental toxins in the establishment of PD^6^,^59^. Although exposure to herbicides, fungicides, and pesticides have been shown to elicit Parkinsonian-like symptoms in various model systems, these environmental factors are not thought to account for all associations between environmental risk and disease^10^,^20^. This study demonstrated that a substantial portion of the cultivable *Streptomyces* community from soil samples has the ability to produce a neurodegenerative compound(s) and may be another mechanism for environmental exposure.

## Methods

### Soil Collection

Approximately 10 g of soil was collected from ~3–5 cm below the surface using a clean garden trowel and placed into a small resealable plastic bag. Each soil sample was thoroughly homogenized. From agricultural soils, samples were obtained between crop rows to avoid damage to the crop and collection of root rhizosphere communities (Fig. 1A). Undeveloped samples were collected from unused land, often requiring that significant leaf litter and debris be removed to access the soil surface, while urban samples were taken from soil near roadways, parking lots and large businesses (Fig. 1B,C). Additional soil (~1.5 liters) was collected from each site and placed into a resealable plastic bag for preparation of an environmental-specific medium and measurement of edaphic properties. Following collection, soil was stored at 4 °C until use. Soil samples were analyzed for particle size distribution, organic content, and pH. Particle size distribution within soils was assessed using a dried sieving method. Approximately 2 g of air-dried soil samples were sieved using U.S. standard mesh sizes #230,#60,#35, and #10. Sieves were shaken at a constant rate of 1100 rpm for 20 minutes using a titer plate shaker (Lab Line Instruments). Based on retention on the sieves, particles were classified as large grain sand, medium grain sand, fine grain sand, or clay/silt. A Bouyoucos hydrometer (VWR International) was employed to further differentiate between clay and silt. For this analysis, 50 g of air-dried soil samples were shaken at 270 rpm overnight on a C1 platform shaker (New Brunswick Scientific) in 50 mL of 5% sodium hexametaphosphate in water solution. Prior to analysis, the samples were diluted to 1 L with deionized water, mixed for 30 seconds, and readings of specific gravity were taken at 40 seconds and 2 hours. A control solution, consisting of 50 mL 5% sodium hexametaphosphate diluted to 1 L, was used to account for variation in specific gravity resulting from the dispersing solution. Following drying of soil at 100 °C overnight, soil organic content was determined by ashing 1 g of soil at 500 °C overnight in a muffle furnace (Thermo Scientific). Samples were weighed to 10^−4^ gram to determine carbon loss and measured in triplicate. Soil pH was measured on 10 g soil in 10 mL deionized water using a waterproof handheld pH meter (Oakton).

### Bacterial Cultivation

Soil suspensions were prepared by adding 0.25 g of soil from the small plastic bag to 1.0 mL of sterile deionized water, mixing thoroughly by vortexing, and serially diluting the suspension to 10^−2^ and 10^−3^. From each dilution, 75 μL was plated in duplicate onto actinobacterial*-*specific and environment-specific media. Mannitol-soy agar^60^ supplemented with 1% sodium pyruvate and starch casein agar (1.5% bacteriological agar, 1% soluble starch, 1% sodium pyruvate, 0.2% potassium phosphate dibasic, 0.2% potassium nitrate, 0.2% sodium chloride, 0.03% casein, 0.005% MgSO_4_+7 H_2_O, 0.002% CaCO_3_, 0.002% FeSO_4_+7H_2_O in 1 L deionized water) were used for their high selectivity for Actinobacteria. To make environment specific media, 1 liter of each soil sample was mixed with 1 liter of deionized water, incubated at 4 °C overnight, and the supernatant was filtered with cheesecloth to remove debris and large sand particles. Bacteriological agar (1.5%) and sodium pyruvate (1%) were added to the filtered supernatant prior to autoclaving. All media were supplemented with cycloheximide and naladixic acid at concentrations of 25 μg/ml to inhibit the growth of fungi and Gram-negative bacteria, respectively, as well as sodium pyruvate to reduce the occurrence and impact of reactive oxygen species^61^. Over a six-week incubation at room temperature, the distinctive colonial morphology (e.g., dry, wrinkled, powdery) for which *Streptomyces* are known was used to select colonies for isolation.

### Molecular Methods

DNA was extracted by suspending cells from isolates in 100 μL of a sterile 5% Chelex (analytical grade 100 resin; Bio-Rad Laboratories) in deionized water solution followed by 4 freeze/thaw cycles with periodic vigorous vortexing. Isolates were verified as members of the genus *Streptomyces* through PCR amplification using *Streptomyces*-specific primers, StrepB (5′-ACAAGCCCTGGAAACGGGGT-3′) and StrepF (5′-ACGTGTGCAGCCCAAGACA-3′)^62^. Each reaction contained 25 pmol of each primer, 10 μL PerfectTaq buffer (5 Prime), 0.125 μg MgCl_2_, 100 mM of each deoxynucleoside triphosphate, 2 U PerfectTaq DNA Polymerase (5 Prime), 1 μL template DNA, and sterile deionized water to a final volume of 50 μL. Reaction conditions were 5 min at 95 °C, followed by 30 cycles of 45 sec at 95 °C, 40 sec at 50 °C, and 120 sec at 72 °C, with a final 10 min incubation at 72 °C. PCR products were analyzed by electrophoresis at 80 V on 1.5% agarose gels. Gels were stained with GelRed™ (Biotium) and visualized under UV transillumination with a gel imaging system (Fotodyne Inc.).

Isolates confirmed as streptomycetes by a positive amplification with *Streptomyces*-specific primers were subjected to BOX PCR^38^ using the BOXA1R primer. The PCR reaction mixtures consisted of 0.3 μg BoxA1R primer (5′-CTACGGCAAGGCGACGCTGACG-3′), 10 μL 5x Green Go *Taq* Buffer (Promega), 0.125 μg MgCl_2_, 4 μL DMSO, 100 mM of each deoxynucleoside triphosphate, 5 U Go *Taq* DNA Polymerase (Promega), 3 μL template DNA, and sterile deionized water to a final volume of 50 μL. Reaction conditions were 7 min at 95 °C, followed by 30 cycles of 90 °C for 30 sec, 53 °C for 60 sec and 65 °C for 8 min, with a final 16 min incubation at 65 °C. PCR products were analyzed by electrophoresis on 1.5% agarose gels at 80 V for 85 minutes. Gels were stained with GelRed™ (Biotium) and visualized under UV transillumination with a gel imaging system (Fotodyne Inc.).

### Growth and isolation of environmental samples for neurodegenerative testing

*Streptomyces* isolates from collection locations representing each soil classification (agricultural, undeveloped, and urban) and all of the major physiographic provinces within the state were selected haphazardly. Each isolate was inoculated into 10 mL of SYZ broth^63^ and incubated at 30^o^  C with shaking at 220 rpm for 17–21 days. In total, 60 isolates from each land use were grown for testing. Samples were harvested approximately 14 days after maximum cell density had been reached. Cell debris was removed by centrifugation at 10,000 x g for 10 minutes and supernatants were sequentially passed through three PES filter membranes with pore sizes of 1 μm, 0.45 μm and 0.22 μm. After the final filtration, the supernatant of each sample was dispensed into five equal aliquots and frozen for subsequent experiments. Each aliquot was thawed and used only once.

### *Caenorhabditis elegans* strains

Nematodes were maintained using standard procedures^64^. Strain BY200 *vtIs1* [P_*dat-1*_::GFP, pRF4(*rol-6*(*su1006*))] (an integrated P_*dat-1*_::GFP strain), a generous gift from Randy Blakely (Vanderbilt University), was used in all experiments.

### *C. elegans* neurodegeneration assay

Each filtered sample was added to the surface of nematode growth medium (NGM) petri plates at a final concentration of 25 μl/ml along with *E. coli* (strain OP50)^5^. Adult P_*dat-1*_::GFP animals (strain BY200) were placed on the environmental sample plates and allowed to lay eggs for ~4 hours before the adults were removed. The worm embryos were grown under constant exposure to *Streptomyces* supernatants until analysis. Worms were transferred to freshly made plates every other day and scored for neurodegeneration after 12 days of exposure. Similarly, P_*dat-1*_::GFP worms exposed to *S. venezueulae* supernatant or *E. coli* supernatant were used as positive and negative controls, respectively, and were analyzed along with worms exposed to the metabolites from the environmental *Streptomyces* isolates. A total of 20-30 worms were analyzed for each environmental isolate. Worms were considered normal when all six anterior dopaminergic neurons were intact and no visible signs of degeneration were observed. If a worm displayed a neuron with a missing or shortened neuronal process, rounding or cell body loss, or blebbing process, the neuron was scored as exhibiting a degenerative change^5^. Further, the number of abnormal neurons/worm was scored on a scale of 0 to 6, with 0 representing a worm with no visible dopamine neuron degeneration, and 6 signifying that a worm exhibited degeneration in all six anterior dopamine neurons. For example, if a worm lacked one dopaminergic cell body and also had two degenerating dendrites, the worm was assigned a score of 3, signifying that 3 out of 6 neurons showed degeneration. This scale allowed for an assessment of the severity of neuronal damage within a population of neurons. After assigning each worm a score, the scores for all worms exposed to each supernatant were added together to yield the total number of degenerating neurons.

### Statistical Analysis

The densiometric curves of all *Streptomyces* isolates and the 180 tested isolates were statistically evaluated using the software package Gel Compar II (BioSystematica). Each densiometric curve was based on the BOX banding pattern and the intensity of each band in relation to other bands within the sample^38^. To prevent ambiguities associated with size determination, only bands between 300 and 3000 base pairs were used for analyses. Isolates with greater than 90% similarity of densiometric curves were considered clones^39^. To produce a similarity matrix, Pearson’s correlations were calculated using 2% optimization and 1% curve smoothing with negative similarities unchanged from the default value^65^. The cluster analysis was calculated using unweighted pair group-method arithmetic average (UPGMA) with advanced clustering. Similarly, to compare banding patterns for all isolates recovered from a particular sample, composite profiles were generated using the create_averaged_fingerprints script provided by Gel Compar II staff. Comparisons of the profiles were performed using both curve-based (e.g., Pearson) and band-based (e.g., Jaccard) coefficients to generate similarity matrices. These matrices were imported into Primer^66^ and one-way ANOSIM, non-metric multidimensional scaling analyses, and cluster analyses were performed to evaluate if *Streptomyces* communities from particular land uses were similar.

SPSS statistical software (version 19) was used to analyze differences in isolation of *Streptomyces* spp. and neurodegenerative compound-producing species across land use patterns and soil characteristics. Measurements of streptomycete isolation followed a normal distribution; parametric tests including a one-way ANOVA and Pearson correlation were used to measure significance. Measurements of sand, silt, clay, and SOM were log transformed to achieve assumptions of normal distribution and parametric tests such as one-way ANOVA with Tukey’s HSD and Pearson correlation were used to measure significance. Nonparametric tests including the Kruskal-Wallis, Mann Whitney and Spearman correlation were used in analyses involving soil pH. Examination of compound-producing species by land use exhibited a normal distribution and parametric tests including ANOVA and Pearson’s correlation were used in analyses.

To determine which *Streptomyces* isolates caused significant dopaminergic neurodegeneration in *C. elegans* (in comparison to the negative *E. coli* control), a one-way ANOVA followed by a Bonferroni correction was employed (Prism 3.0 software; GraphPad). For analysis of the *Streptomyces* isolates that caused significant neurodegeneration with varying land uses, total numbers of damaged neurons were compared using a one-way ANOVA followed by Tukey’s HSD.

## Additional Information

**How to cite this article**: Watkins, A. L. *et al*. The Prevalence and Distribution of Neurodegenerative Compound-Producing Soil *Streptomyces* spp. *Sci. Rep.* **6**, 22566; doi: 10.1038/srep22566 (2016).

## Supplementary Material

Supplementary Information

## Figures and Tables

**Table 1:** Average number of distinct *Streptomyces* isolates (based on BOX PCR banding patterns) and standard deviation from diluted 0.25 g soil samples across land uses and physiographic provinces.

	Agricultural	Undeveloped	Urban	*Average*
Appalachian Plateaus	22.1 ± 8.8 (6)	14.2 ± 8.6 (6)	23.3 ± 8.3 (4)	*19.4* ± *9.1 (16)*
Piedmont Province	22.2 ± 11.4 (5)	11.8 ± 7.8 (6)	23.8 ± 6.9 (5)	*18.8* ± *9.9 (16)*
Valley and Ridge	38.0 ± 1.1 (1)	14.5 ± 8.8 (4)	18.0 ± 10.6 (4)	*18.7* ± *11.3 (9)*
Black Belt Prairies	30.3 ± 8.5 (4)	13.3 ± 8.3 (3)	24.3 ± 14.4 (4)	*22.6* ± *12.2 (14)*
Coastal Plains	20.1 ± 10.3 (7)	18.9 ± 10.5 (11)	16.5 ± 5.7 (6)	*18.7* ± *9.3 (25)*
Interior Low Plateau	27.5 ± 9.9 (4)	18.3 ± 11.9 (3)	10.0 ± 0.0 (2)	*20.6* ± *11.2 (9)*
*Average*	*24.1* ± *10.0 (27)*	*15.1* ± *9.2 (33)*	*20.0* ± *9.1 (25)*	

Sample sizes are shown in parentheses.
