# Supplementary material for: The Prevalence and Distribution of Neurodegenerative Compound-Producing Soil Streptomyces spp
Source: Sci Rep. 2016 Mar 3;6:22566. doi: 10.1038/srep22566 (PMC4776106; doi:10.1038/srep22566)
Supplement: Supplementary Information [file srep22566-s1.pdf]

The Prevalence and Distribution of Neurodegenerative Compound-Producing Soil *Streptomyces* spp.

Anna L. Watkins, Arpita Ray, Lindsay Reynolds, Kim A. Caldwell, Julie B. Olson

Supplemental information on sampling locations throughout the state of Alabama and soil characteristics at each site; AG = agriculture; UD = undeveloped; UR = urban; SOM = soil organic matter; ND = not determined

Latitude	Longitude	Physiographic Region	Physiographic province	Land Use	Estimated Streptomyces g/soil	soil pH	SOM (mg/g)	% SOM
30.436	-87.598	Atlantic Plains	Coastal Plains	AG	1.30E+07	7.31	30.566	2.877
30.406	-87.683	Atlantic Plains	Coastal Plains	UR	8.00E+06	5.83	45.804	4.411
30.407	-87.691	Atlantic Plains	Coastal Plains	UR	1.20E+07	6.17	58.379	5.432
31.737	-85.684	Atlantic Plains	Coastal Plains	AG	3.00E+08	6.79	45.226	4.400
31.664	-85.668	Atlantic Plains	Coastal Plains	UD	1.20E+07	6.57	32.577	3.156
32.952	-87.137	Atlantic Plains	Coastal Plains	UD	2.20E+08	7.32	62.417	6.324
32.930	-87.067	Atlantic Plains	Coastal Plains	UR	1.20E+07	7.57	70.013	7.085
32.895	-86.989	Atlantic Plains	Coastal Plains	UD	8.90E+06	6.09	56.424	4.518
33.930	-86.793	Appalachian Highlands	Appalachian Plateaus	UD	2.20E+08	6.59	86.646	7.852
32.143	-85.715	Atlantic Plains	Coastal Plains	UD	2.50E+08	5.5	103.170	10.116
32.302	-86.012	Atlantic Plains	Coastal Plains	UR	2.30E+08	7.25	72.887	7.023
32.734	-85.588	Appalachian Highlands	Piedmont	UD	1.80E+06	6.66	69.128	6.670
33.274	-85.875	Appalachian Highlands	Piedmont	UD	2.50E+08	5.15	97.277	9.554
33.274	-85.838	Appalachian Highlands	Piedmont	UR	2.80E+08	7.86	57.882	5.411
33.146	-85.967	Appalachian Highlands	Piedmont	UD	1.20E+07	5.06	ND	ND
33.142	-85.969	Appalachian Highlands	Piedmont	UR	1.40E+07	7.84	45.418	3.871
31.609	-85.889	Atlantic Plains	Coastal Plains	AG	2.80E+08	5.17	14.541	1.339
31.608	-85.883	Atlantic Plains	Coastal Plains	UD	1.70E+07	5.11	32.964	3.163
32.882	-86.244	Appalachian Highlands	Piedmont	AG	1.30E+07	6.11	66.873	6.262
32.881	-86.241	Appalachian Highlands	Piedmont	UD	2.60E+06	5.05	38.119	3.238
32.891	-86.218	Appalachian Highlands	Piedmont	UR	1.50E+07	6.81	85.647	7.688
32.881	-86.246	Appalachian Highlands	Piedmont	AG	2.90E+08	5.94	68.182	5.850
34.050	-86.778	Appalachian Highlands	Appalachian Plateaus	UD	8.90E+06	6.53	148.201	14.929
34.162	-86.836	Appalachian Highlands	Appalachian Plateaus	UR	1.10E+07	7.03	334.729	32.986
34.262	-86.937	Appalachian Highlands	Appalachian Plateaus	AG	2.10E+08	6.95	27.426	2.268
31.615	-85.724	Atlantic Plains	Coastal Plains	UD	3.00E+08	5.64	60.611	5.157
32.439	-87.349	Atlantic Plains	Black Belt Prairies	AG	2.40E+08	7.55	136.535	13.556
32.439	-87.342	Atlantic Plains	Black Belt Prairies	UD	1.30E+07	5.51	99.191	9.301

32.439	-87.319	Atlantic Plains	Black Belt Prairies	AG	2.00E+08	5.22	141.007	15.799
34.568	-85.612	Appalachian Highlands	Appalachian Plateaus	AG	2.70E+08	7.17	116.264	12.175
34.541	-85.605	Appalachian Highlands	Valley and Ridge	UD	2.00E+08	5.87	165.584	18.081
32.468	-86.384	Atlantic Plains	Coastal Plains	AG	1.90E+08	7.38	ND	ND
32.468	-86.384	Atlantic Plains	Coastal Plains	UD	7.10E+06	4.66	25.778	1.926
32.557	-86.009	Atlantic Plains	Coastal Plains	UR	2.10E+08	6.79	91.240	8.170
34.047	-86.181	Appalachian Highlands	Valley and Ridge	AG	3.60E+08	5.89	119.557	12.931
34.047	-86.176	Appalachian Highlands	Valley and Ridge	UD	4.40E+06	5.09	31.752	2.539
33.597	-87.662	Appalachian Highlands	Appalachian Plateaus	UD	2.00E+08	6.11	93.868	9.691
33.650	-87.509	Appalachian Highlands	Appalachian Plateaus	AG	1.20E+07	5.91	38.862	3.255
34.371	-87.577	Appalachian Highlands	Appalachian Plateaus	AG	2.20E+08	6.32	87.267	7.053
34.365	-87.563	Appalachian Highlands	Appalachian Plateaus	UD	1.90E+08	5.08	50.425	4.779
33.002	-87.627	Atlantic Plains	Coastal Plains	UD	1.20E+07	5.85	50.578	4.242
32.704	-87.592	Atlantic Plains	Coastal Plains	UR	2.10E+08	5.8	60.228	5.571
33.354	-86.988	Appalachian Highlands	Valley and Ridge	UD	2.20E+08	7.09	107.919	12.022
33.392	-86.988	Appalachian Highlands	Valley and Ridge	UR	2.70E+08	7.32	61.914	6.681
33.354	-86.988	Appalachian Highlands	Valley and Ridge	UR	1.50E+07	6.8	114.662	12.183
34.484	-87.424	Interior Plains	Interior Low Plateaus	AG	1.50E+07	5.7	45.545	3.575
34.492	-87.212	Interior Plains	Interior Low Plateaus	UD	2.80E+08	7.81	53.769	4.905
32.691	-85.481	Appalachian Highlands	Piedmont	AG	8.00E+06	5.02	41.818	3.430
33.216	-85.939	Appalachian Highlands	Piedmont	UD	1.90E+08	7.98	57.349	4.482
32.535	-85.486	Appalachian Highlands	Piedmont	UR	2.30E+08	7.59	58.703	4.361
34.688	-86.974	Interior Plains	Interior Low Plateaus	AG	3.40E+08	6.69	60.068	5.549
34.665	-86.948	Interior Plains	Interior Low Plateaus	AG	4.20E+08	6.46	56.388	4.922
32.333	-85.849	Atlantic Plains	Black Belt Prairies	AG	2.80E+08	6.58	28.359	2.743
32.333	-85.849	Atlantic Plains	Black Belt Prairies	UR	1.80E+07	7.68	115.209	10.366
34.943	-86.571	Interior Plains	Interior Low Plateaus	AG	2.20E+08	7.57	59.447	6.604
34.928	-86.572	Interior Plains	Interior Low Plateaus	UD	1.10E+07	6.86	72.757	6.015
34.928	-86.572	Interior Plains	Interior Low Plateaus	UR	8.90E+06	7.6	97.578	7.224
32.312	-86.047	Atlantic Plains	Black Belt Prairies	AG	4.10E+08	7.03	116.139	10.281
32.312	-86.047	Atlantic Plains	Black Belt Prairies	UD	2.30E+08	7.17	98.187	9.049

32.309	-86.047	Atlantic Plains	Black Belt Prairies	UR	2.80E+08	6.69	156.664	14.133
32.343	-86.221	Atlantic Plains	Black Belt Prairies	UR	7.10E+06	7.67	74.343	7.377
34.591	-87.052	Interior Plains	Interior Low Plateaus	UD	9.80E+06	7.36	ND	ND
34.603	-86.987	Interior Plains	Interior Low Plateaus	UR	8.90E+06	7.6	99.677	9.355
32.449	-87.513	Atlantic Plains	Black Belt Prairies	UD	1.80E+07	8.06	104.294	8.738
32.449	-87.473	Atlantic Plains	Black Belt Prairies	UR	3.90E+08	7.68	149.334	15.283
31.632	-85.749	Atlantic Plains	Coastal Plains	AG	2.80E+08	6.52	17.976	1.733
31.632	-85.751	Atlantic Plains	Coastal Plains	UD	9.80E+06	5.18	37.113	3.235
33.561	-86.148	Appalachian Highlands	Valley and Ridge	UD	8.00E+06	6.2	69.890	5.600
33.503	-86.160	Appalachian Highlands	Valley and Ridge	UR	4.40E+06	4.9	92.681	8.633
33.437	-86.090	Appalachian Highlands	Valley and Ridge	UR	2.70E+08	7.94	59.435	5.628
32.911	-85.775	Appalachian Highlands	Piedmont	AG	1.80E+07	4.5	84.477	7.417
32.835	-85.759	Appalachian Highlands	Piedmont	UD	1.30E+07	5.4	77.186	7.393
32.908	-85.776	Appalachian Highlands	Piedmont	UR	2.90E+08	7.93	44.146	3.647
32.953	-86.005	Appalachian Highlands	Piedmont	AG	3.60E+08		93.439	9.780
33.283	-87.623	Atlantic Plains	Coastal Plains	AG	1.70E+07	5.86	60.268	5.548
33.283	-87.624	Atlantic Plains	Coastal Plains	UD	2.90E+08	5.69	58.604	5.380
33.283	-87.623	Atlantic Plains	Coastal Plains	UD	3.40E+08	5.94	54.905	4.887
33.502	-87.606	Atlantic Plains	Coastal Plains	AG	8.90E+06	5.93	65.869	4.908
33.961	-87.608	Appalachian Highlands	Appalachian Plateaus	UD	7.10E+06	5.38	97.252	11.242
33.872	-87.246	Appalachian Highlands	Appalachian Plateaus	AG	8.90E+06	6.49	51.833	3.602
33.871	-87.254	Appalachian Highlands	Appalachian Plateaus	UR	2.20E+08	5.73	71.142	6.210
33.720	-87.384	Appalachian Highlands	Appalachian Plateaus	UR	2.80E+08	7.25	113.389	9.256
33.849	-87.285	Appalachian Highlands	Appalachian Plateaus	UR	2.70E+08	6.51	210.271	21.638
34.146	-87.494	Appalachian Highlands	Appalachian Plateaus	AG	2.80E+08	5.91	42.749	3.253
34.093	-87.574	Appalachian Highlands	Appalachian Plateaus	UD	2.60E+06	6.68	ND	ND
